# Chloroplast genome structure and phylogenetic position of *Ruppia sinensis* Shuo Yu & den Hartog

**DOI:** 10.1080/23802359.2019.1662746

**Published:** 2019-09-05

**Authors:** Shuo Yu, Miao-Miao Shi, Kai Jiang

**Affiliations:** a Fourth Institute of Oceanography, Ministry of Natural Resources, Beihai, China;; b Key Laboratory of Plant Resources Conservation and Sustainable Utilization, South China Botanical Garden, Chinese Academy of Sciences, Guangzhou, China;; c Shanghai Chenshan Botanical Garden, Shanghai, China;; d School of Ecological and Environmental Sciences, Shanghai Key Lab of Urban Ecological Processes and Eco-Restoration, East China Normal University, Shanghai, China

**Keywords:** *Ruppia sinensis*, Illumina sequencing, chloroplast genome, phylogenetic position

## Abstract

*Ruppia* is widely distributed in marine and inland saline habitats in temperate and tropical regions. In this study, the complete chloroplast genome sequence of *R. sinensis* was successfully obtained using Illumina sequencing. The full length of the chloroplast genome length was 158,897 bp with a typical quadripartite structure: one large single copy (LSC) region (88,952 bp), one small single copy (SSC) region (19,047 bp), and a pair of inverted repeats (IR) (25,449 bp each). The GC content of this genome was 35.9%. The whole genome contained 136 genes, including 88 protein-coding genes, 40 tRNA genes, and eight rRNA genes. Phylogenetic analysis indicated that *R. sinensis* formed a distinct clade, being separated from *Zostera marina* and *Potamogeton perfoliatus*.

Plants of the genus *Ruppia* are distributed in temperate and tropical regions worldwide, even beyond the polar circle (den Hartog and Kuo 2006). It occurs in permanent and temporary bodies of water with the salinity ranging from fresh to 230‰ total dissolved solutes (Brock 1982). *Ruppia* plants are very simple but show great variation in morphology and are characterized by strongly branched stems and slender leaves. Because of the high intraspecific phenotypic plasticity and hybridization, the taxonomy of this genus has been contested (Triest and Sierens 2010; Yu and den Hartog 2014). Nevertheless, using the chloroplast DNA data combined with morphological traits is a very powerful means of identifying *Ruppia* species (Yu et al. 2014). In this study, we sequenced the complete chloroplast genome of *Ruppia sinensis* using next-generation technology. We expect this genome will be very useful in taxonomic studies of *Ruppia*.

Fresh *R. sinensis* plants were collected from an abandoned saltern in Yancheng, Jiangsu Province, China (33.41°N, 120.28°E) and the voucher specimen was deposited at Fourth Institute of Oceanography Herbarium (YC201905-1). Genomic DNA was extracted from the cleaned shoots using a modified CTAB method and then sequenced using the Illumina Novaseq platform. Low-quality reads and adapters were trimmed off using the FastQC software (Andrews 2010). De novo genome assembly was conducted with SPAdes v3.9 (Bankevich et al. 2012). The complete chloroplast genome was annotated using DOGMA (Wyman et al. 2004). The annotations of tRNA genes were made using ARAGORN (Laslett and Canback 2004). The annotated complete chloroplast genome of *R. sinensis* was submitted to the GenBank database (Accession Number: MN233650).

The complete cp genome sequence of *R. sinensis* was 158,897 bp in length with a characteristic circular structure, including a pair of inverted repeats (IRs) (25,449 bp), one large single-copy region (88,952 bp), and one small single-copy region (19,047 bp). The guanine-cytosine (GC)-content was 35.9%. There was a total of 136 genes in this genome, consisting of 88 protein-coding genes, 40 tRNA genes, and eight rRNA genes. There were 20 duplicated genes in the IRs regions, eight protein-coding genes (rpl2, rpl23, ycf2, ycf15, ndhB, rps7, rps12, and ycf1), eight tRNA genes (trnI-CAU, trnL-CAA, trnV-GAC, trnI-GAU, trnA-UGC, trnR-CCG, trnR-ACG, and trnN-GUU), and four rRNA genes (rrn16, rrn23, rrn4.5, and rrn5).

To establish the phylogenetic position of *R. sinensis*, we then downloaded 23 completed chloroplast genomes from the GenBank database (Figure 1). The whole cp genomes were aligned with MAFFT (Katoh and Standley 2013) and the phylogenetic trees were reconstructed using RAxML software (Stamatakis 2014) with maximum likelihood method. As shown in the phylogenetic tree, *Ruppia sinensis* formed a distinct clade, being separated from *Zostera marina* and *Potamogeton perfoliatus*.

**Figure 1.**
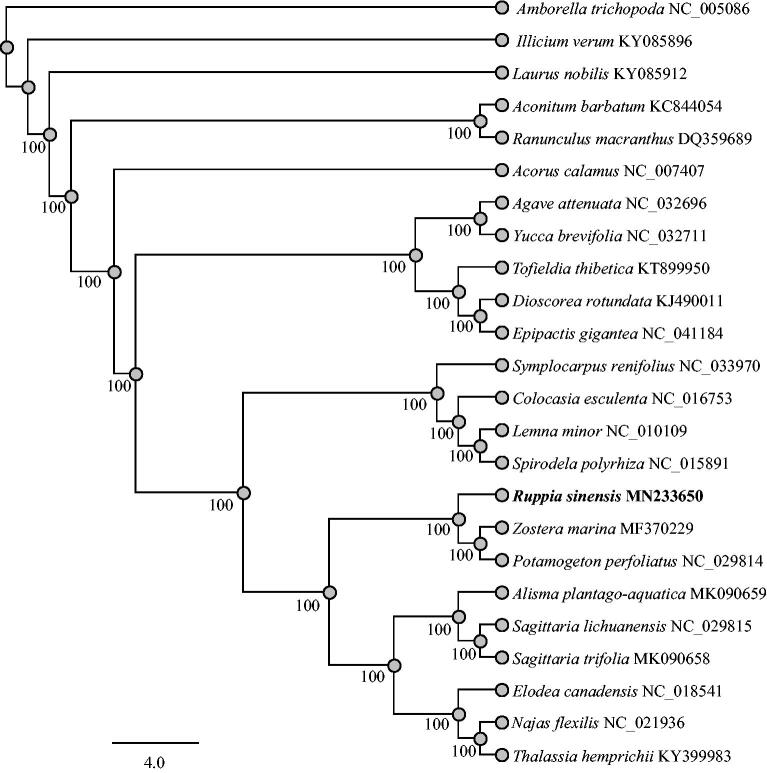
Phylogenetic relationship of 24 species based on the chloroplast genome sequences with Maximum likelihood (ML) analysis.
